# Late Onset Ipilimumab-Induced Pericarditis and Pericardial Effusion: A Rare but Life Threatening Complication

**DOI:** 10.1155/2015/794842

**Published:** 2015-03-30

**Authors:** Seongseok Yun, Nicole D. Vincelette, Iyad Mansour, Dana Hariri, Sara Motamed

**Affiliations:** ^1^Department of Medicine, University of Arizona, Tucson, AZ 85721, USA; ^2^Molecular Pharmacology and Experimental Therapeutics, Mayo Clinic, Rochester, MN 55905, USA; ^3^Department of Pathology, University of Arizona, Tucson, AZ 85721, USA; ^4^Midwestern University, Arizona College of Osteopathic Medicine, Glendale, AZ 85308, USA

## Abstract

Metastatic cutaneous melanoma has poor prognosis with 2-year survival rate of 10–20%. Melanoma cells express various antigens including gp100, melanoma antigen recognized by T cells 1 (MART-1), and tyrosinase, which can induce immune-mediated anticancer response via T cell activation. Cytotoxic T-lymphocyte associated antigen-4 (CTLA-4) is an immune check point molecule that negatively regulates T cell activation and proliferation. Accordingly, recent phase III clinical trials demonstrated significant survival benefit with ipilimumab, a human monoclonal antibody (IgG1) that blocks the interaction of CTLA-4 with its ligands. Since the efficacy of ipilimumab depends on T cell activation, it is associated with substantial risk of immune mediated adverse reactions such as colitis, hepatitis, thyroiditis, and hypophysitis. We report the first case of late onset pericarditis and cardiac tamponade associated with ipilimumab treatment in patient with metastatic cutaneous melanoma.

## 1. Case Presentation

A 59-year-old male patient with no significant history of autoimmune disease presented to clinic with bleeding from a mole in the right forearm. Biopsy and mutation testing identified melanoma with BRAF^V600E^ mutation. PET/CT showed four FDG avid soft tissue nodules in the subcutaneous tissues of chest and back, abdominal mesentery, and right retroperitoneum. Excisional biopsy from right axillary lymph node was positive for melanin A staining and showed extracapsular invasion, confirming the diagnosis of stage M1c metastatic melanoma. Therefore, patient received 4 cycles of ipilimumab (3 mg/kg) treatment every 3 weeks without significant adverse reaction except skin rash on the infusion site.

**Figure 1:**
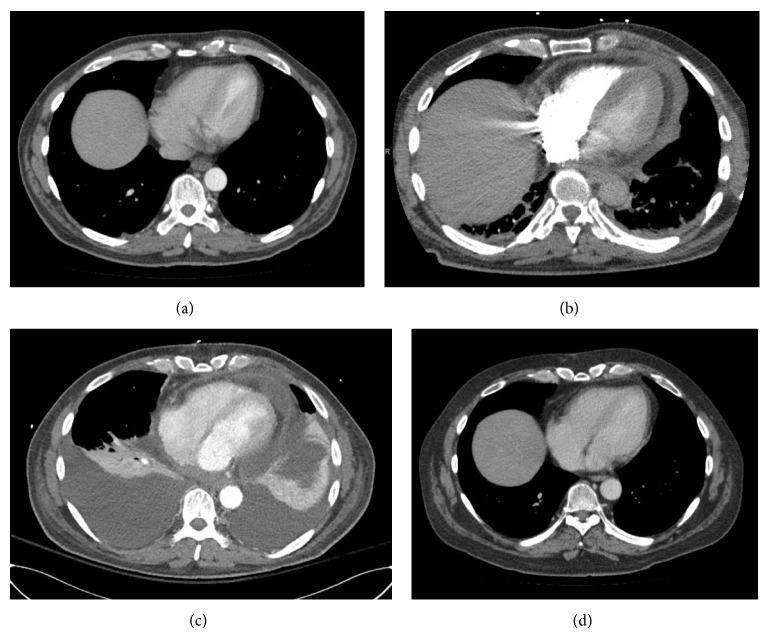
CT chest on admission showed pericardial thickening, moderate-sized pericardial effusion, and adjacent inflammatory changes within the epicardial fat and mediastinum (b), which were new compared to the prior study (a). New large bilateral pleural effusion with associated compressive atelectasis in the lower lungs and stable pericardial effusion is observed on day 10 (c), which resolved after systemic steroid treatment (d).

**Figure 2:**
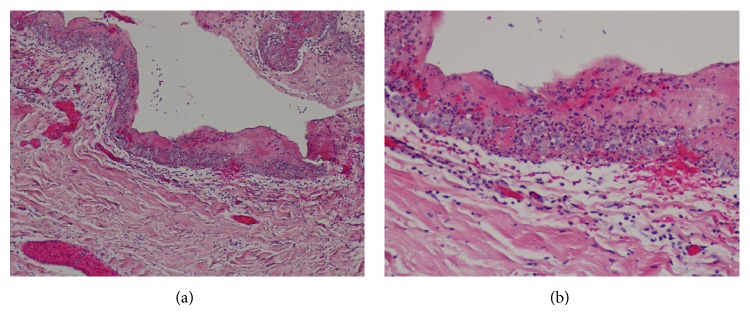
Biopsy specimen from pericardium, sections through the pericardium show acute fibrinous pericarditis, characterized by mixed inflammatory infiltrates in the pericardial wall, accompanied by abundant surface fibrin. No microorganisms were identified on hematoxylin-eosin-stained sections.

Twelve weeks after the last cycle of ipilimumab treatment, the patient presented to ED with acute onset chest pain and shortness of breath which started 1 day prior to the presentation. Vital sign showed BP 97/55 mmHg, HR 106 beats/min, RR 20 breaths/min, and O_2_ saturation 99% while breathing room air and temperature 36.9°C. Physical examination revealed distant heart sound and 5 cm of jugular venous distension. Electrocardiogram showed low QRS voltage and T wave inversion on V_1_–V_4_ leads, and troponin I was negative. CT angiogram showed negative for pulmonary embolism; however, it demonstrated pericardial thickening and moderate sized pericardial effusion which are new compared to the prior study (Figures 1(a) and 1(b)). Subsequent echocardiogram showed septal bouncing and respiratory septal shift, suggesting ventricular interdependence and constrictive effusive physiology. Total 3 L of fluid was given for low blood pressure. Bedsides pericardiocentesis drained 130 mL of serosanguinous fluid and subxiphoid pericardial window was performed the next day. Biochemical study from pericardial fluid showed LDH 794 IU/L, protein 4.3 g/dL, amylase 29 IU/L, and glucose 99 mg/dL. Fluid cytology, Gram stain, and culture were negative for neoplasm or microorganism, and adenosine deaminase PCR was also negative. WBC count was 19,600/*μ*L with 90% of lymphocyte consistent with marked acute inflammation. Pathology from pericardial tissue demonstrated acute fibrinous pericarditis without any evidence of malignancy or microorganism (Figure 2). Additional examinations for autoimmune disease including rheumatoid factor, anti-nuclear antibodies (ANA), double strand DNA (dsDNA), anti-neutrophil cytoplasmic antibodies (ANCA), proteinase 3, and myeloperoxidase antibodies were all negative. Further infectious work-up including blood culture, sputum culture, and respiratory viral panel were all negative as well.

Indomethacin (50 mg three times a day) was started for the treatment of acute pericarditis; however, patient developed worsening shortness of breath, generalized weakness, somnolence, and diarrhea. Blood pressure dropped down to 64/42 for which levophed and aggressive fluid resuscitation was initiated. Repeat CT scan demonstrated persistent pericardial effusion and large bilateral pleural effusion with compressive atelectasis in the lower lobes (Figure 1(c)). Thoracentesis was performed to drain 1.4 L of pleural fluid and biochemistry revealed borderline exudates with LDH ratio 0.27, protein ratio 0.51, and WBC 667/*μ*L with lymphocyte dominance (57%) but no evidence of malignancy or infection. Brain MRI showed no pathologic changes. TSH, free T4, and morning random cortisol levels after the last cycle of ipilimumab treatment were 3.26 *μ*IU/mL, 0.8 ng/dL, and 10.6 *μ*g/dL, respectively, and rechecked levels on admission showed 6.78 *μ*IU/mL, 0.4 ng/dL, and 1.0 *μ*g/dL, indicating hypothyroidism and adrenal insufficiency (Table 1). Screening colonoscopy prior to ipilimumab treatment had shown normal finding and infectious work-up for the new onset diarrhea including* C. diff* toxin PCR, stool Gram stain, culture, and parasites was all negative. Collectively, these results suggested ipilimumab induced immune-mediated pericarditis, hypothyroidism, adrenal insufficiency, and diarrhea for which high dose intravenous methylprednisolone (125 mg daily) was started. Patient achieved remarkable clinical improvement over the 48 hours, and methylprednisolone was switched to prednisone (40 mg daily) and budesonide (9 mg daily) on the third day, and they were tapered down over a month. Repeat chest X-ray and CT scan showed resolved pleural and pericardial effusion (Figure 1(d)), and diarrhea improved gradually over the month. Rechecked TSH and random cortisol levels also showed normal range of 2.85 *μ*IU/mL (without thyroid hormone replacement) and 1.5 *μ*g/dL, respectively (Table 1).

## 2. Discussion

Over the past decades, the incidence of cutaneous melanoma has increased by more than 60%, and 10–15% of patients present at stage III or IV [1]. Unresectable disease is used to be treated with best supportive care, radiation, or systemic treatments such as dacarbazine and temozolomide; however, the prognosis of metastatic disease is dismal with median survival of less than 12 months [2, 3]. Immune system plays a pivotal role to eradicate cancer cells, making immune modulation a novel therapeutic target. Recognition of various tumor antigens by antigen presenting cells induces cytotoxic T cell activation via interaction of T cell receptor with major histocompatibility complex 1 molecule. For the full T cell activation, additional engagement of costimulatory pathway is required and this is antagonized by CTLA-4, an immune check point molecule. CTLA-4 competes with CD28 to bind CD80 (B7-1) and CD86 (B7-2) and negatively regulates T cell activation and proliferation [4, 5]. Accordingly, CTLA-4 knockout mice were shown to develop lymphoproliferative disorder with excessive accumulation of activated T cells and preclinical study with antibodies against CTLA-4 demonstrated tumor cells suppression [6, 7].

Ipilimumab is a human monoclonal antibody (IgG1) that blocks the interaction of CTLA-4 with its ligands and recent phase III clinical trials in patients with unresectable metastatic melanoma showed overall survival benefit with ipilimumab treatment compared to gp100 vaccination or dacarbazine monotherapy [8, 9]. Because antagonizing CTLA-4 stimulates T cell proliferation, ipilimumab treatment is associated with substantial risk of immune mediated adverse reactions and current guideline recommends ipilimumab treatment with careful monitor for these side effects [10]. Previous phase II and III clinical trials showed that grade 3-4 immune related adverse events including enterocolitis, hepatitis, dermatitis, and endocrinopathy can occur in 10–40% of patients [5, 8, 9, 11–13] and rare complications such as pericarditis, nephritis, pneumonitis, meningitis, uveitis, and hemolytic anemia in less than 1% of patients who were treated with ipilimumab (Table 2) [14].

The halflife of ipilimumab clearance is 14.7 days [14]; however, immune cell activation and proliferation are slow process [15]. Accordingly, the effect of ipilimumab treatment evolves over months and delayed responses and adverse events (18–20 weeks after treatment) are well known as in this case [15]. Our patient completed the last cycle of ipilimumab treatment 12 weeks prior to admission and he presented with pericarditis and pericardial effusion. Infectious work-up including bacterial and viral etiologies was negative. There was no significant history of autoimmune disease and additional examinations for autoimmune disease were all negative. Moreover, pericardial and pleural fluids cytology showed lymphocytes dominance with no evidence of malignancy or infection and pericardial tissue biopsy demonstrated acute inflammation, suggesting ipilimumab induced immune mediated pericarditis and pericardial effusion, most likely. This is supported by associated hypothyroidism, adrenal insufficiency, and diarrhea, all of which showed remarkable improvement with systemic steroid treatment and without hormone replacement.

As shown in phase II and III clinical trials, most of the ipilimumab induced immune related adverse effects are reversible with early recognition and appropriate management. For the severe immune reactions, early administration of high dose systemic corticosteroid is critical and adverse reactions resolve within a median of 2-3 weeks as in our case [5, 8, 9, 11]. Collectively, ipilimumab treatment is associated with significant survival benefit and, however, also with life threatening immune mediated adverse effects that require close monitor, early diagnosis, and appropriate management.

## 3. Conclusion

To the best of our knowledge, this is the first case of late onset pericarditis and pericardial effusion associated with ipilimumab treatment in patient with metastatic cutaneous melanoma. Ipilimumab induced immune mediated adverse events could be life threatening as shown in our case, and early diagnosis and intervention with systemic corticosteroid are critical for the better clinical outcome.

## Figures and Tables

**Table 1:** Lab values during the hospitalization, baseline TSH, free T4, and cortisol levels were within normal range; however, patient presented with elevated TSH, low free T4, and decreased cortisol levels, suggesting immune-mediated hypothyroidism and adrenal insufficiency.

Labs (reference range)	Baseline	Admission	Day 7 after steroid Tx	Day 21 after steroid Tx	Day 35 after steroid Tx
TSH (0.35–4.00 *μ*IU/mL)	3.26	6.78	8.10	5.85	2.85
Free T4 (0.7–1.5 ng/dL)	0.8	0.4	0.4	1.2	1.0
ACTH (7–69 pg/mL)	NR	NR	<5	<5	<5
Cortisol at 8 AM (4.2–38.4 *μ*g/dL)	10.6	<1.0	<1.0	<1.0	1.5
AST (5–34 IU/L)	28	30	24	18	21
ALT (0–55 IU/L)	13	14	28	33	19
Total bilirubin (0.2–1.2 mg/dL)	1.1	1.9	0.8	0.6	0.6
Troponin I (0.00–0.02 ng/mL)	NR	<0.02	0.07	<0.02	NR

**Table 2:** Ipilimumab induced immune related adverse events in Phases II and III trials.

Study	Pathology	Stage	Range of median Age	Pt. No.	Treatments	Overall survival rate			Grade 3/4 immune related adverse events rate		
						Ipilimumab (1)	Ipilimumab (2)	Ctrl	Ipilimumab (1)	Ipilimumab (2)	Ctrl
Robert et al., 2011 [9]	Cutaneous melanoma	III IV	56.4–57.5	502	Ipilimumab (10 mg/kg) + dacarbazine (1) versus placebo + dacarbazine	47.3% (1 yr) 28.5% (2 yr) 20.8% (3 yr)		36.3% (1 yr) 17.9% (2 yr) 12.2% (3 yr)	^†^Any events: 41.7% dermatologic: 3.2% GI: 5.6% hepatic: 30%		Any events: 6.0% dermatologic: 0% GI: 0% hepatic: 1.2%

Hodi et al., 2010 [8]	LHA-A^*^0201 (+) cutaneous melanoma	III IV	55.6–57.4	676	Ipilimumab (3 mg/kg) + gp100 (1) versus ipilimumab (3 mg/kg) (2) versus gp100	43.6% (1 yr) 21.6% (2 yr)	45.6% (1 yr) 23.5% (2 yr)	25.3% (1 yr) 13.7% (2 yr)	^‡¶^Any events: 10.2% dermatologic: 2.4% GI: 5.8% hepatic: 3.2% endocrine: 1.1% others: 1.3%	Any events: 14.5% dermatologic: 1.5% GI: 7.6% hepatic: 0% endocrine: 3.8% others: 2.3%	Any events: 3.0% dermatologic: 0% GI: 0.8% hepatic: 2.3% endocrine: 0% others: 3.1%

Wolchok et al., 2010 [11]	Cutaneous melanoma	III IV	56–59	217	Ipilimumab (10 mg/kg) (1) versus ipilimumab (3 mg/kg) (2)	48.6% (1 yr) 29.8% (2 yr)	39.6% (1 yr) 24.2% (2 yr)		^¶^Any events: 25.4% dermatologic: 4.2% GI: 15.5% hepatic: 3.0% endocrine: 1.4% ^*δ*^others: 2.8%	Any events: 7.0% dermatologic: 1.4% GI: 2.8% hepatic: 0% endocrine: 2.8% others: 0%	

Hersh et al., 2011 [13]	Cutaneous melanoma	III IV	60–66	76	Ipilimumab (3 mg/kg) + dacarbazine (1) versus ipilimumab (3 mg/kg) (2)	62.0% (1 yr) 24.0% (2 yr) 20.0% (3 yr)	45.0% (1 yr) 21.0% (2 yr) 9.0% (3 yr)		^*Ф*^Any events: 17.1% dermatologic: 42.9% GI: 28.6%	Any events: 7.1% dermatologic: 48.7% GI: 20.5%	

Weber et al., 2009 [12]	Cutaneous melanoma	III IV	58–61	115	Ipilimumab (10 mg/kg) + placebo (1) versus ipilimumab (10 mg/kg) + budesonide (2)	62.4% (1 yr) 41.7% (2 yr)	55.9% (1 yr) 40.5% (2 yr)		^¶^Any events: 38.0% dermatologic: 0% GI: 23.0% hepatic: 12.0% endocrine: 5.0% others: 2.0%	Any events: 41.0% dermatologic: 5.0% GI: 24.0% hepatic: 9.0% endocrine: 5.0% others: 2.0%	

^†^The immune-related adverse events were prospectively defined (medical dictionary for regulatory activities, version 13.0).

^‡^The immune-related adverse events were defined as an adverse event that was associated with exposure to the study drug and that was consistent with an immune phenomenon.

^¶^The adverse events were graded by the National Cancer Institute's common terminology criteria for adverse events version 3.0.

^*δ*^Other immune related adverse events included scleritis (*n* = 1) and pneumonitis (*n* = 1).

^*Ф*^Immune related adverse events were coded according to the Medical Dictionary for Regulatory Affairs, and severities were graded using Common Toxicity Criteria version 2.0. Dermatologic and GI adverse events included grade ≥1 in this study.
